# Comprehensive molecular analyses of an autoimmune-related gene predictive model and immune infiltrations using machine learning methods in intracranial aneurysma

**DOI:** 10.3389/fimmu.2025.1531930

**Published:** 2025-04-17

**Authors:** Minxue Zhang, Lin Zhou, Yuying Zhao, Yanling Wang, Zhuobo Zhang, Zhan Liu

**Affiliations:** Department of Neurology, The Fourth Affiliated Hospital of Harbin Medical University, Harbin, China

**Keywords:** intracranial aneurysma, machine learning, bioinformatics, immune infiltration, autoimmune-related genes

## Abstract

**Background:**

Increasing evidence indicates a connection between intracranial aneurysm (intracranial aneurysm, IA) and autoimmune diseases. However, the molecular mechanisms from a genetic perspective remain unclear. This study aims to elucidate the potential roles of autoimmune-related genes (ARGs) in the pathogenesis of IA.

**Methods:**

Three transcription profiles (GSE13353, GSE26969, and GSE75436) for intracranial aneurysm (IA) were obtained from GEO databases. Autoimmune-related genes (ARGs) were sourced from the Genecards databases. Differentially expressed ARGs (DEARGs) were identified using the “limma” R package. GO, KEGG and GSEA analyses were performed to uncover underlying molecular functions. Three machine learning methods—LASSO logistic regression, random forest (RF), and XGBoost—were employed to identify key genes. An artificial neural network was used to develop an autoimmune-related signature predictive model for IA. Immune characteristics, including immune cell infiltration, immune responses, and HLA gene expression in IA, were investigated using ssGSEA. Additionally, the miRNA-gene regulatory network and potential therapeutic drugs for hub genes were predicted. In certain sections of the written content of this manuscript, the authors have utilized text generated by an AI technology. The specific name, version, model, and source of the generative AI technology used are as follows: Generative AI Technology Name: ChatGPT, Version: 4.0, Model: GPT-4, Source: OpenAI.

**Results:**

A total of 39 differentially expressed ARGs (DEARGs) were identified across the GSE13353, GSE26969, and GSE75436 datasets. From these, two key diagnostic genes were identified using three machine learning algorithms: ADIPOQ and IL21R. A predictive neural network model was developed based on these genes, exhibiting strong diagnostic capability with a ROC value of 0.944, and further validated using a nomogram approach. The study focused on intracranial aneurysm (IA), revealing significant insights into the underlying genetic mechanisms.

**Conclusion:**

The results of bioinformatics analysis in our study elucidated the mechanism of intracranial aneurysm (IA), identifying two key differential genes. Our research highlights the significant roles of immune infiltration and the regulatory networks between genes, miRNAs, and drugs in IA. These findings not only enhance our understanding of the pathogenesis of IA but also suggest potential new avenues for its treatment.

## Introduction

Intracranial aneurysms (IA) are localized dilatations of cerebral arteries, posing a significant risk of rupture and subsequent subarachnoid hemorrhage (SAH), a life-threatening condition with high morbidity and mortality (1, 2). While research has traditionally focused on structural and hemodynamic aspects of IA development, the potential role of immune responses in IA formation and progression has been increasingly recognized (3).

Inflammation is considered an important aspect of IA pathophysiology (4). Recent studies have revealed a heightened risk of IA in individuals with autoimmune diseases (5, 6),such as systemic lupus erythematosus and rheumatoid arthritis, suggesting a potential link between autoimmune responses and IA pathogenesis (7, 8).

Autoimmune-related genes (ARGs) are integral to the regulation of various immune responses, including inflammation, immune cell activation, and apoptosis, all of which could contribute to IA development (9). Immune complexes, a hallmark of autoimmune disorders, can trigger vasculitis, leading to narrowed or occluded vessels, a process implicated in IA formation (10). However, the precise role and mechanisms of ARGs in IA pathogenesis remain poorly understood.

To delve deeper into the role of ARGs in IA, this study utilizes a comprehensive bioinformatics approach to analyze differentially expressed ARGs (DEARGs) using three transcriptome datasets (GSE13353, GSE26969, and GSE75436) obtained from the GEO database. We perform functional enrichment analyses, including Gene Ontology (GO) and Kyoto Encyclopedia of Genes and Genomes (KEGG) pathway analyses, to uncover the biological processes and pathways involving DEARGs. Furthermore, we employ three machine learning methods, LASSO logistic regression, random forest (RF), and XGBoost, to identify key DEARGs that could serve as potential diagnostic biomarkers for IA. An artificial neural network model is constructed based on these key genes to predict IA risk and further validated using a nomogram approach.

To elucidate the immune landscape of IA, we investigate immune cell infiltration, immune responses, and HLA gene expression using single-sample gene set enrichment analysis (ssGSEA). Additionally, we predict miRNA-gene regulatory networks and potential therapeutic drugs targeting hub genes, providing novel insights into potential therapeutic interventions for IA.

This study aims to comprehensively analyze the role of ARGs in IA pathogenesis and develop an ARG-based predictive model for IA. Our findings may provide novel insights into the pathophysiology of IA and pave the way for the development of new diagnostic and therapeutic strategies.

## Materials and methods

### Data acquisition

The gene expression proffling data of aneurysm samples GSE13353, GSE26969 (11), and GSE75436 (GPL570 platform),was obtained from the NCBI GEO database (https://www.ncbi.nlm.nih.gov/geo/) and was retrieved by our team. The datasets include 37 intracranial aneurysm patients and 18 age- and gender-matched control samples of normal superficial temporal artery. If multiple probes matched the same gene, the probe with the highest median expression value was annotated to the corresponding homologous gene symbol through the platform’s annotation information.

Normalization was performed on the raw GEO data via the “NormalizeBetweenArray” R package. It was determined that the R utility known as “Limma” was the best tool for analyzing Differentially Expressed Genes (DEGs) comparing normal and sample samples. P-values that were either <0.05 or were equal to 0.05 itself were regarded as statistically significant. In order to determine whether genes displayed differential expression, we employed the LogFC (log fold change) > 1 and adjusted P 0.05 criteria.

ARGs were obtained from the Genecards database (https://www.genecards.org/) after deleting duplicate genes. Filter relevance socre>3.

### Identification of differentially expressed autoimmune-related genes

We conducted a principal component analysis (PCA) using the factoextra package in R to explore the data structure. To identify differentially expressed DEARGs, we utilized the limma package in R to perform differential expression analysis. This allowed us to detect differentially expressed genes (DEGs) between the intracranial aneurysm (IA) and control groups. DEGs were identified based on the criteria of |log2FoldChange| > 1 and a p-value < 0.05. For visualization, volcano plots and clustering heatmaps were generated using the ggplot2 and ComplexHeatmap packages, respectively. To identify DEARGs, we intersected the identified DEGs with aging-related genes (ARGs) and visualized the overlap using the VennDiagram package in R.

### PPI network analysis

Leveraging the full capabilities of the STRING website facilitated the creation of a graphical representation of PPIs, encompassing 39 nodes and 138 edges. Subsequently, this network was imported into the Cytoscape application for further analysis.

### Gene ontology and Kyoto encyclopedia of genes and genomes analysis

We performed a comprehensive analysis of the identified genes using widely recognized bioinformatics tools and databases to explore their functional implications and potential involvement in critical biological processes related to intracranial aneurysms (IA). Utilizing the clusterProfiler R package (v4.0), we conducted Gene Ontology (GO) and Kyoto Encyclopedia of Genes and Genomes (KEGG) pathway enrichment analyses to gain valuable insights into the biological functions and pathways associated with these genes. GO analysis allowed us to systematically categorize and annotate the genes based on their associated biological processes, molecular functions, and cellular components. By applying rigorous GO analysis, we aimed to uncover the fundamental biological processes and functions these genes contribute to within the context of IA development and progression. This detailed ontological classification provided us with a comprehensive understanding of the molecular intricacies underlying this complex disease. Additionally, we employed the KEGG database to unravel the intricate network of signaling pathways that these genes participate in. By performing KEGG pathway enrichment analysis with a significance threshold of P < 0.05, we identified crucial biological pathways significantly enriched among these genes. This analysis was essential for comprehending the interconnectedness of these genes within specific cellular processes and molecular cascades relevant to IA, offering valuable insights into potential dysregulation and perturbations during IA development and suggesting potential therapeutic targets and strategies.

We conducted principal component analysis using the factoextra R package. Differential expression analysis was performed with the limma package in R to detect DEGs between the AI and control groups in datasets GSE13353, GSE26969, and GSE75436. DEGs were screened with the criteria of |log2FoldChange| > 1 and p < 0.05. Volcano plots and clustering heatmaps were prepared to visualize the differences using the ggplot2 and Complex Heatmap packages in R. We intersected the DEGs with ARGs to identify DEARGs and visualized them with the VennDiagram package.

### Feature selection of characteristic biomarkers via three machine learning methods

## Results

In order to identify characteristics that could serve as diagnostic biomarkers for IA, we employed LASSO, XGBoost, and RF methodologies. To develop the LASSO model, we utilized the glmnet package, incorporating tenfold cross-validation along with a tuning/penalty parameter to optimize feature selection. This process helped to identify a preliminary set of potentially relevant features. XGBoost is a powerful gradient boosting machine learning algorithm, was employed to further refine and enhance the feature set identified by LASSO. Specifically, XGBoost’s ability to capture complex interactions and non-linear relationships within the data allowed us to identify features that might have been missed by LASSO alone. This approach also helped to improve the overall predictive performance of the model. The RF approach, a randomization algorithm designed to prevent overfitting of individual decision trees, was implemented. RF leverages multiple decision trees derived from the same training set to enhance model performance and provide a robust estimate of feature importance.

**Figure 1:**
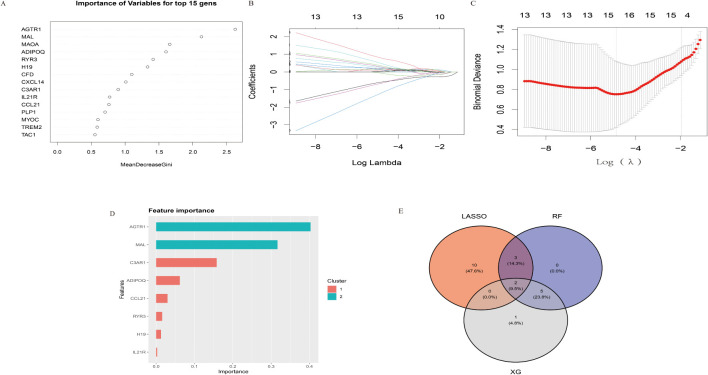
Identification of key diagnostic biomarkers using LASSO, XGBoost, and Random Forest (RF) methodologies. **(A)** Importance of variables determined by the Random Forest method, shown for the top 15 genes using the MeanDecreaseGini index. **(B)** Coefficient paths for the LASSO model across varying log lambda values. **(C)** Cross-validation results for the LASSO model, displaying binomial deviance against log(lambda) values. **(D)** Feature importance as determined by the XGBoost algorithm. **(E)** Venn diagram illustrating the intersection of selected genes from LASSO, XGBoost, and RF methodologies. Two genes were consistently identified across all three methods.

To identify a set of robust and potentially clinically relevant biomarkers, we intersected the features identified by each of the three methods. This process, visualized in the Venn diagram (**Figure 1E**), involved identifying the genes that were consistently selected as important by LASSO, XGBoost, and RF. We identified two diagnostic genes by overlapping the three algorithms (**Figure 1E**): The detailed descriptions of the two diagnostic signatures are listed in **Table 1**.

**Table 1:** Detail information about the two hub genes identified by machine learning.

Gene	Description	Chromosome	logFC	P.Value	Change
ADIPOQ	Adiponectin, C1Q And Collagen Domain Containing	3	-2.825	1.82×10^−9^	DOWN
IL21R	Interleukin 21 Receptor	16	1.662	4.29×10^−7^	UP

### Identification of differentially expressed autoimmune-related genes

We utilized Gene Set Variation Analysis (GSVA) with the R package GSVA to estimate gene set enrichment variation, focusing on immune cell types. Box plots were generated to compare infiltration levels between control and IA groups, and statistical significance was assessed using [specify test, e.g., t-test or Wilcoxon rank-sum test], with significance levels indicated by asterisks (*p < 0.05, **p < 0.01, ***p < 0.001). Heatmaps visualized expression changes of selected genes across cell types. Violin plots displayed the distribution of gene expression levels for ADIPOQ and IL-21 R between groups, highlighting differences in expression patterns. ROC curve analysis evaluated the predictive performance of gene expression signatures, with the Area Under the Curve (AUC) calculated to quantify accuracy, where values closer to 1 indicated better discriminative ability.

### Gene set enrichment analysis

The Gene Set Enrichment Analysis (GSEA) was performed on the genes in question with the intention of understanding the biological significance of the distinguishing genes (12). In order to arrive at a normalized enrichment score for each analysis, one thousand distinct gene set permutations have to be done. Reference gene sets included hallmark, c2kegg, c2biocartar and c5go. The screening conditions were |normalized enrichment score (NES)| > 1, nominal (NOM) p-value < 0.05 and FDR q-value < 0.25.

### Construction of an aneurysm-related genetic and nomogram

The R package ‘rms’ (version 6.5.0) was employed to construct a nomogram for predicting the risk of intracranial aneurysms. This involved integrating genetic markers with clinical features identified in the training dataset to generate a comprehensive risk prediction model. The nomogram assigned point values to each variable, which were then summed to provide an overall risk score.

Calibration curves were generated using the R package ‘PredictABEL’ (version 1.2.4) to assess the accuracy of the nomogram. These curves compared predicted probabilities of aneurysm risk with observed outcomes, ensuring the model’s reliability. The ROC curve was used to evaluate the model’s discriminative ability, with the area under the curve (AUC) calculated to quantify performance.

### Construction of regulatory networks

Network Analyst was used to construct the Gene-miRNA interactions, Protein-drug interactions and Protein-chemical interactions based on signature genes.

## Results

### Differential expression analysis

**Figure 2:**
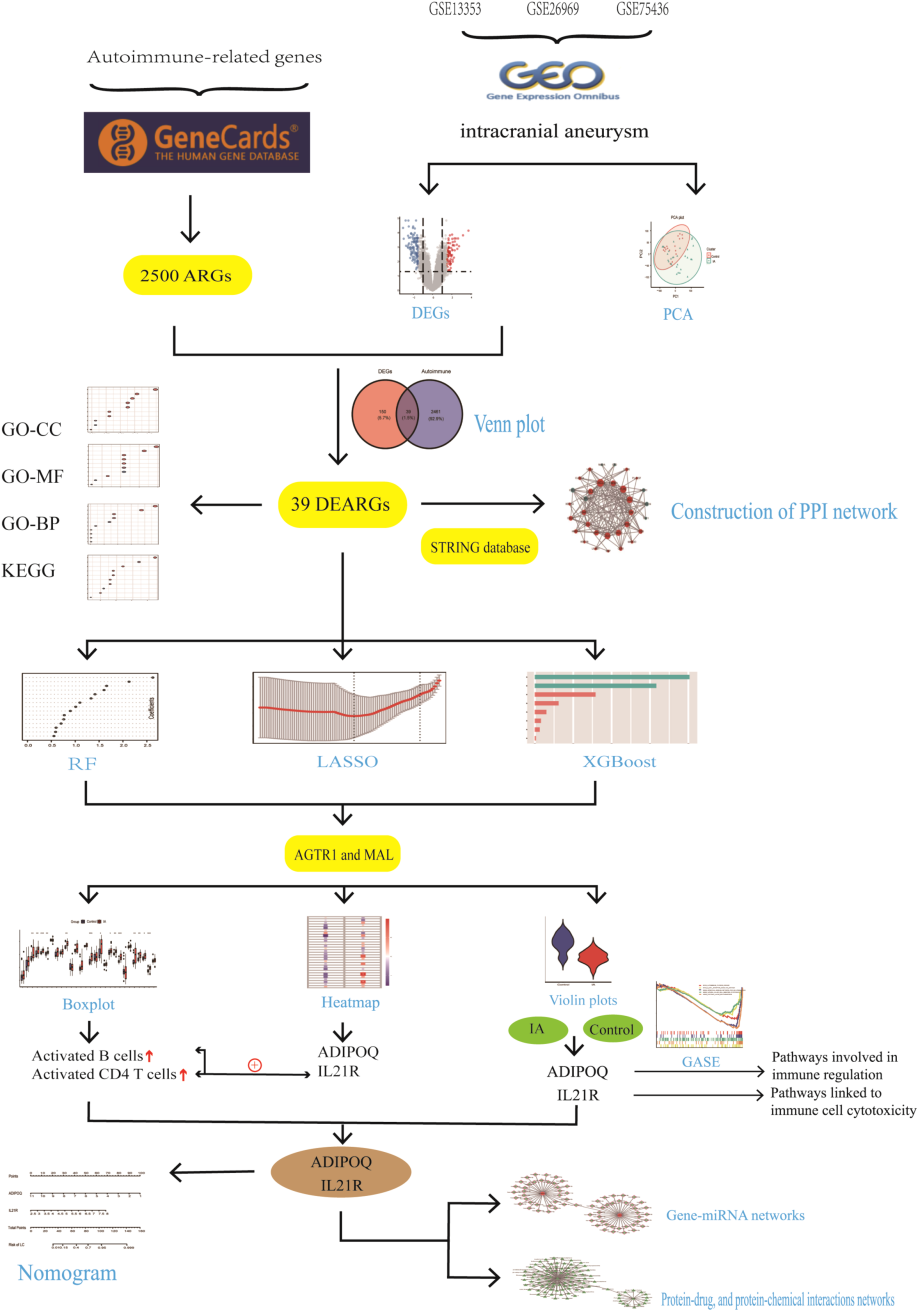
The schematic block diagram of the entire workflow of this study.

**Figure 3:**
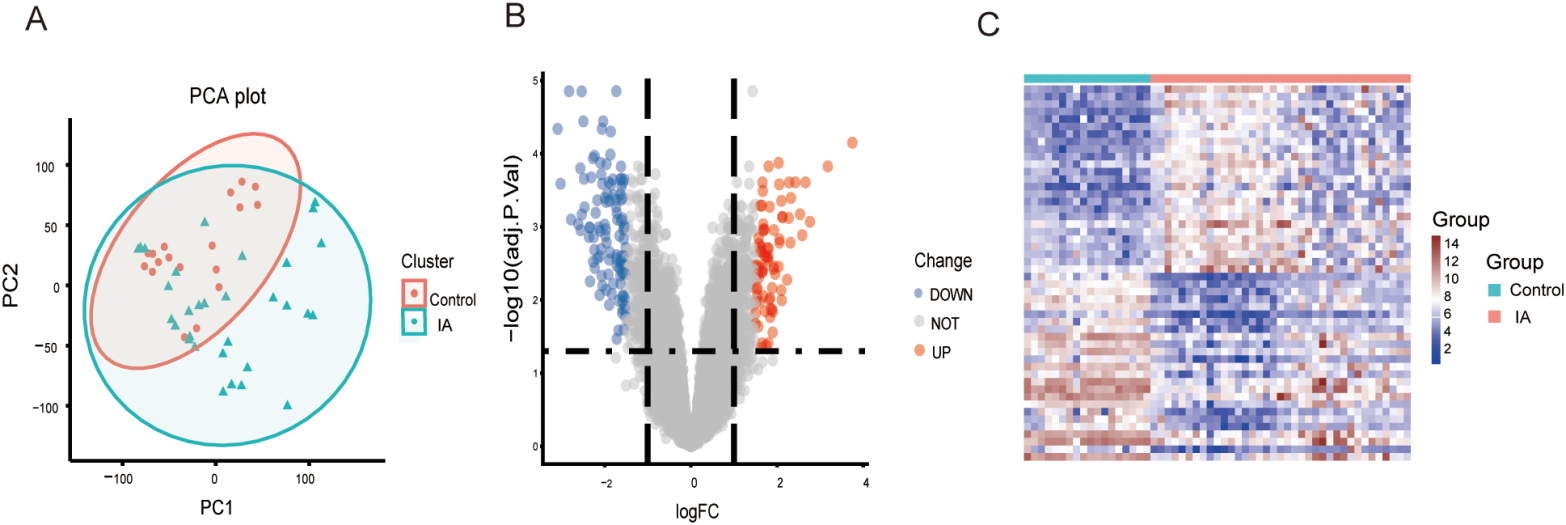
PCA and DEG analysis between IA tissues and controls. **(A)** Principal component analysis between IA tissues and controls. **(B)** A volcano plot shows the DEGs. Blue dots show the down-regulated genes and red dots represent the up-regulated genes. **(C)** A heat map shows the expression patterns of DEGs. PCA, principal component analysis; DEGs, differentially expressed genes; IA, intracranial aneurysma disease.

The study flowchart is depicted in **Figure 2**. Principal component analysis showed that the IA tissues and controls could be clearly distinguished in the GSE13353, GSE26969, and GSE75436 dataset (**Figure 3A**). Differential expression analysis was further performed to screen for DEGs. Based on the selection criteria, 189 DEGs (79 upregulated and 110 downregd) were identified (**Figure 3B**). The expression patterns of these DEGs were visualized through a hierarchical clustering heatmap (**Figure 3C**).

### Construction of gene-gene interaction network of DEARGs

**Figure 4:**
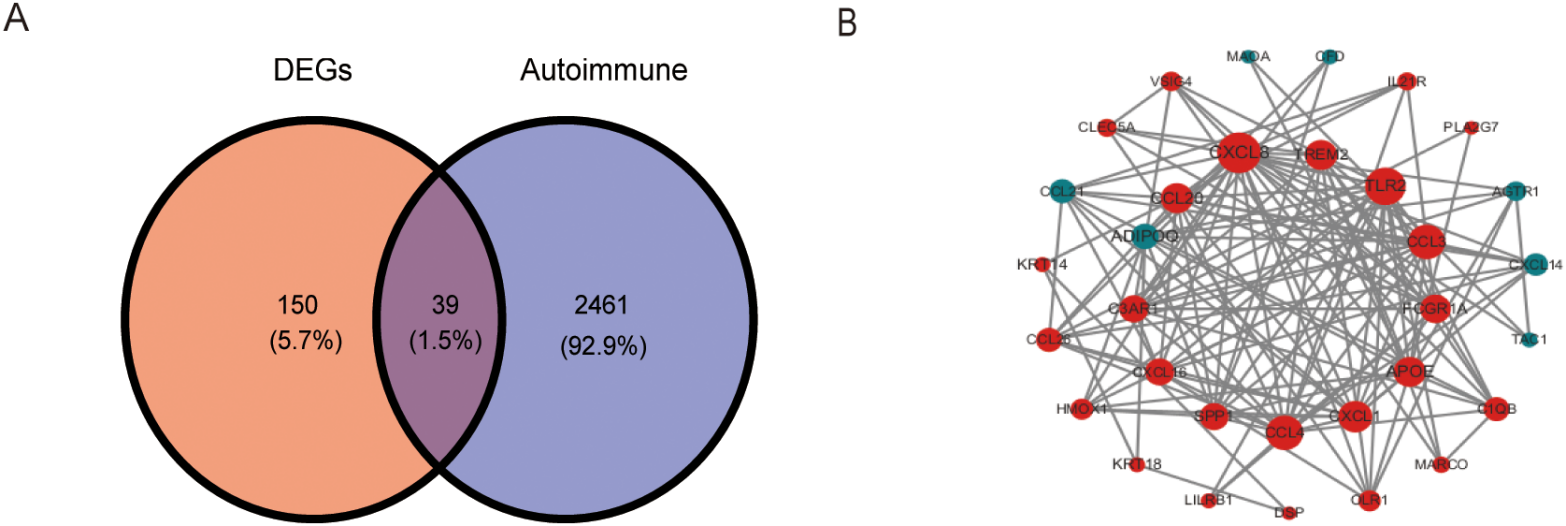
Identification and PPI network construction of DEARGs. **(A)** A venn plot show 189 DEARGs in IA. **(B)** PPI network of DEARGs. The blue nodes represent the down-regulated genes and the red nodes represent the up-regulated genes. The dot size indicates the degree of the nodes. DEARGs, differentially expressed autoimmune-related genes.

We overlapped the DEGs with the 2,500 ARGs obtained from GeneCards, resulting in 189 DEARGs in IA (**Figure 4A**). To elucidate the molecules’ functional associations, we imported these genes into the STRING database to construct a PPI network. The PPI network with 39 nodes and 138 edges was constructed (**Figure 4B**).

### Functional enrichment analysis of DEGs

**Figure 5:**
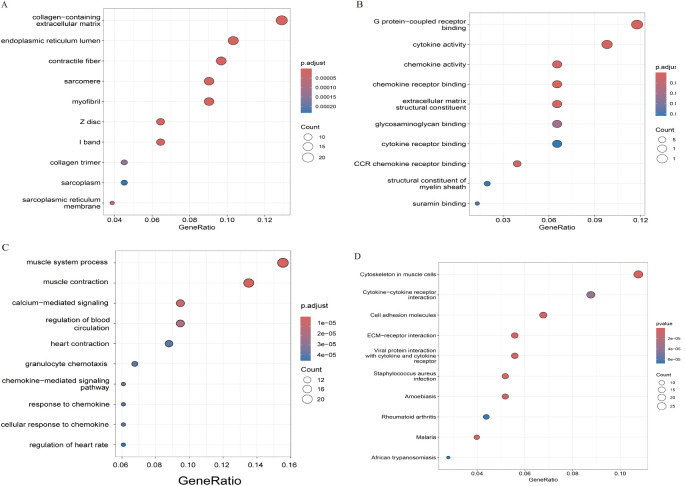
Functional enrichment study based on DEGs, **(A)** analysis of the GO-CC. **(B)** analysis of the GO-MF. **(C)** analysis of the GO-BP. **(D)** KEGG pathway analysis.

The findings of the GO analysis were classified into three categories: biological processes, cellular components, and molecular functions. For intracranial aneurysm (IA) and normal samples, the enrichment of cellular components, such as the endoplasmic reticulum lumen, contractile fiber, and sarcoplasmic reticulum membrane, indicates a strong focus on muscle and structural components (**Figure 5A**). This suggests significant alterations in cellular architecture and stress response mechanisms associated with IA. In terms of molecular functions, differentially expressed genes (DEGs) show enrichment in activities such as G protein-coupled receptor binding, cytokine and chemokine activity, and components of the extracellular matrix (**Figure 5B**). These functions are essential for signaling pathways and cellular communication, which are frequently disrupted in IA.

Enrichment is highlighted in biological processes related to the muscle system, such as muscle contraction and regulation of blood circulation (**Figure 5C**). Additionally, processes like calcium-mediated signaling and chemokine-mediated pathways are prominent. These findings suggest that alterations in vascular muscle function and inflammatory responses play a critical role in the pathophysiology of intracranial aneurysms. The dysregulation of these processes may contribute to structural weaknesses and inflammatory environments that predispose individuals to aneurysm formation and progression. Understanding these pathways provides valuable insights into potential therapeutic targets for managing intracranial aneurysms. The enrichment of pathways such as cytokine-cytokine receptor interaction, ECM-receptor interaction, and the role of the cytoskeleton in muscle cells is evident (**Figure 5D**). Additional pathways, including viral protein interactions with cytokines, rheumatoid arthritis, and various infections, highlight the complex interplay between immune responses and the pathology of intracranial aneurysms. These findings underscore the multifaceted nature of IA pathogenesis, involving both structural and immune-related mechanisms.

### Construction and validation of Random Forest, the lasso model and XGBoost, Random Forest methodologies

During our study, we employed multiple powerful machine learning methodologies to identify key diagnostic biomarkers for IA. Using the Random Forest (RF) approach (**Figure 1A**), we assessed the significance of various genes, identifying the top 15 with the highest MeanDecreaseGini scores, reflecting their substantial contribution to the prediction model. Through the LASSO model (**Figure 1B**), we examined coefficient paths across different log lambda values. As regularization increased, the coefficients of less significant features shrank toward zero, refining the feature set. We conducted tenfold cross-validation in the LASSO analysis (**Figure 1C**), selecting the optimal lambda where the binomial deviance was minimized, thus balancing model simplicity and accuracy. The XGBoost algorithm (**Figure 1D**) offered an additional perspective on feature importance. We found that genes such as AGTR1 and MAL were consistently highlighted across two clusters, emphasizing their potential relevance in diagnosing IA.

Finally, by intersecting the features identified by LASSO, XGBoost, and RF, as shown in the Venn diagram (**Figure 1E**), we pinpointed two genes consistently selected by all three methods. These genes, representing the intersection set, may serve as robust and clinically relevant biomarkers for IA. Our comprehensive analyses, integrating multiple machine learning techniques, underscore the significant potential of these biomarkers for further investigation and characterization.

### Immunological analysis of aneurysm

**Figure 6:**
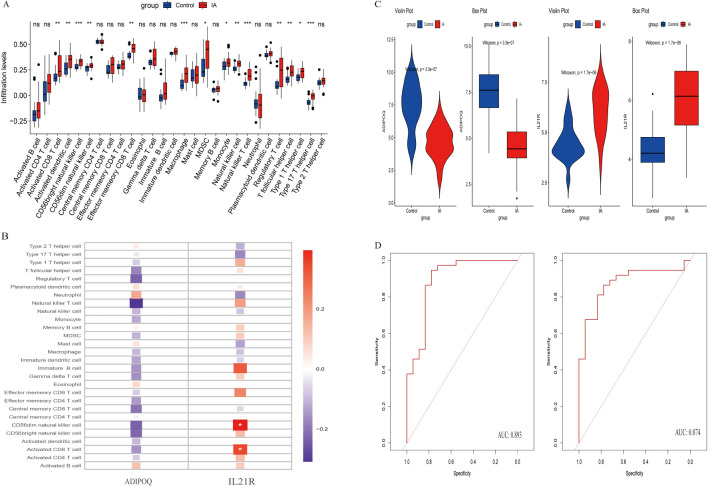
Analysis of immune cell infiltration and gene expression in control and IA groups. **(A)** Box plots show the infiltration levels of various immune cell types in control (blue) and IA (red) groups. Statistical significance is indicated by asterisks (*p < 0.05, **p < 0.01, ***p < 0.001). **(B)** Heatmap illustrating the expression changes of selected genes across different immune cell types. Expression levels range from low (blue) to high (red). **(C)** Violin plots depicting the distribution of gene expression levels for ADIPOQ and IL21R in control and IA groups, emphasizing differences in expression patterns. **(D)** ROC curves assessing the predictive performance of gene expression signatures, with Area Under the Curve (AUC) values indicating model accuracy.

In our immunological analysis of aneurysm samples, we employed several techniques to identify key genes associated with aneurysm development. We conducted a boxplot analysis (**Figure 6A**) comparing immune cell infiltration levels between control and IA groups. Significant differences were observed in various immune cell populations, such as increased infiltration of activated B cells, central memory CD4 T cells, and regulatory T cells in the IA group, indicating altered immune responses in IA. A heatmap analysis **(Figure 6B**) was used to visualize correlations between immune cell types and the expression levels of candidate genes, ADIPOQ and IL21R, which emerged as potential biomarkers due to their distinct expression patterns in IA samples. IL21R showed positive correlations with activated CD4 T cells and activated B cells, highlighting its potential role in the immune landscape of aneurysms. Violin plots (**Figure 6C**) illustrated the expression differences of ADIPOQ and IL21R between control and IA groups. Both genes showed significantly higher expression levels in the IA group, suggesting their potential roles in the pathophysiology of aneurysms. Receiver operating characteristic (ROC) curves (**Figure 6D**) were generated to evaluate the diagnostic potential of ADIPOQ and IL21R. Both genes demonstrated high specificity and sensitivity, with area under the curve (AUC) values indicating strong diagnostic performance for IA. The detailed descriptions of the six diagnostic signatures are listed in **Table 1**. These analyses collectively highlight ADIPOQ and IL12-R as critical genes in aneurysm pathology and potential targets for further investigation and therapeutic intervention.

### Estimation of IA immune cell infiltration

**Figure 7:**
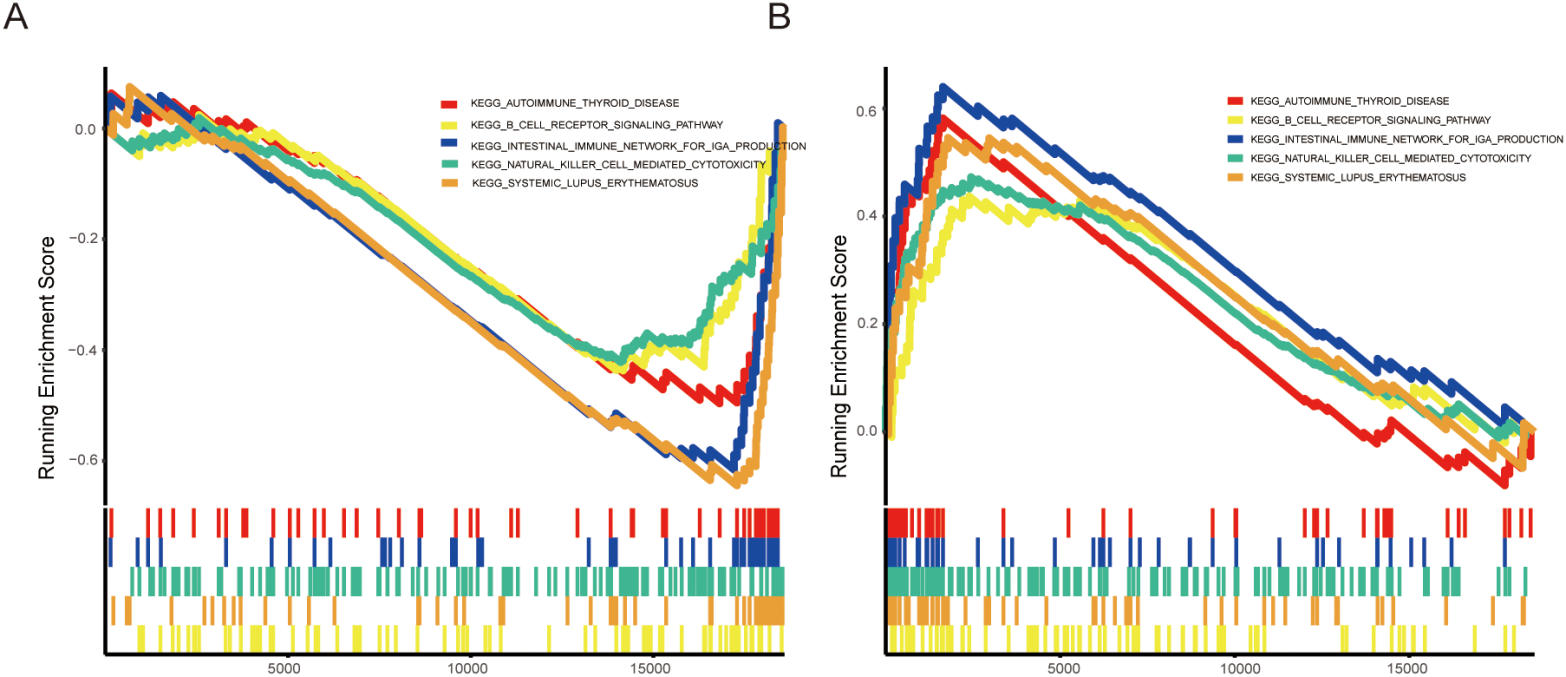
Enrichment Analysis of ADIPOQ and IL21R in IA. **(A)** Enrichment plots for the ADIPOQ gene, highlighting significant enrichment in pathways associated with immune functions, chemokine activity, and MHC molecules. **(B)** Enrichment plots for the IL21R gene, showing strong associations with pathways related to immune response, including chemokine signaling and MHC molecule activity.

In our study, we performed a gene set enrichment analysis (GSEA) to investigate the roles of ADIPOQ and IL21R in aneurysm pathophysiology, focusing on their involvement in immune-related pathways. The GSEA results (**Figure 7A**) revealed that ADIPOQ is significantly associated with pathways involved in immune regulation, including B cell receptor signaling and autoimmune thyroid disease. These findings suggest that ADIPOQ may modulate immune responses in the aneurysm microenvironment, potentially influencing inflammatory and autoimmune processes. By affecting these pathways, ADIPOQ could play a crucial role in the progression or stabilization of aneurysms. Similarly, the enrichment plot for IL21R (**Figure 7B**) shows a strong association with pathways linked to immune cell cytotoxicity, such as those involving natural killer cells and systemic lupus erythematosus.IL21R may enhance cytotoxic and inflammatory responses, suggesting its involvement in immune surveillance mechanisms within aneurysms. This could contribute to both protective and pathological immune activities in the disease context.

### Construction of a nomogram for prognostic prediction

**Figure 8:**
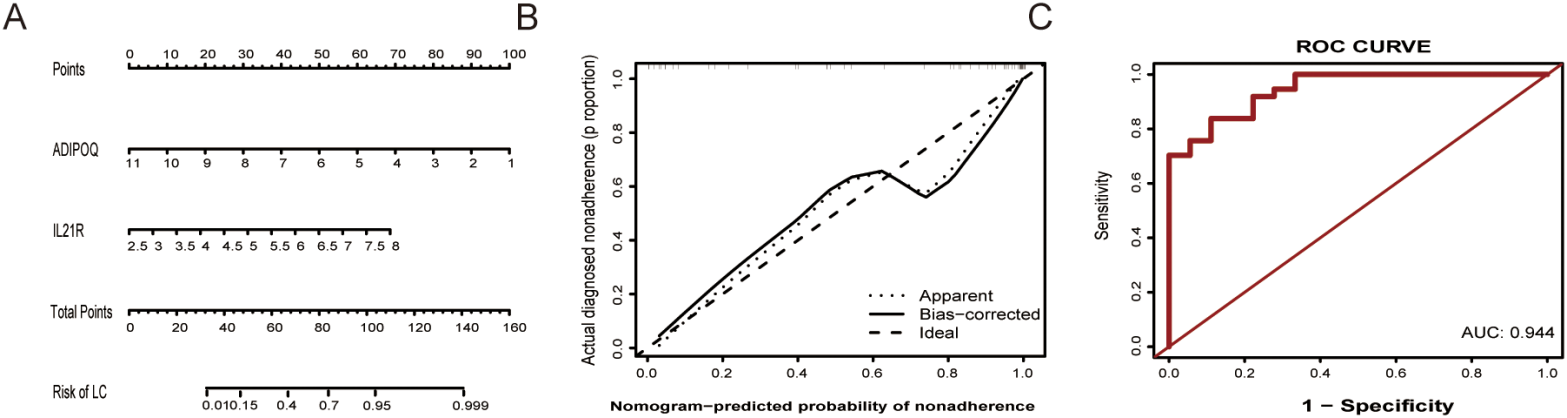
Nomogram and validation for predicting aneurysm risk. **(A)** Nomogram for Aneurysm Risk Prediction: This nomogram integrates the expression levels of ADIPOQ and IL21R to estimate the risk of aneurysm development. **(B)** Calibration Curve shows high accuracy; predicted probabilities closely match actual outcomes on the ideal line. **(C)** ROC Curve illustrates the diagnostic performance of the nomogram, with an area under the curve (AUC) of 0.944.

In our study, a nomogram was constructed to predict the risk of aneurysm development using key biomarkers ADIPOQ and IL21R (**Figure 8A**). This nomogram was designed to estimate the probability of aneurysm occurrence based on individual patient profiles. The calibration curve (**Figure 8B**) demonstrated excellent agreement between the predicted probabilities and actual outcomes. The apparent and bias-corrected lines closely aligned with the ideal line, indicating the nomogram’s accuracy in predicting aneurysm risk. The ROC curve (**Figure 8C**) further validated the nomogram’s predictive performance, with an area under the curve (AUC) of 0.944. This high AUC value signifies strong specificity and sensitivity, underscoring the nomogram’s robustness as a diagnostic tool.

### Establishment of miRNA-gene and drug gene regulatory networks

**Figure 9:**
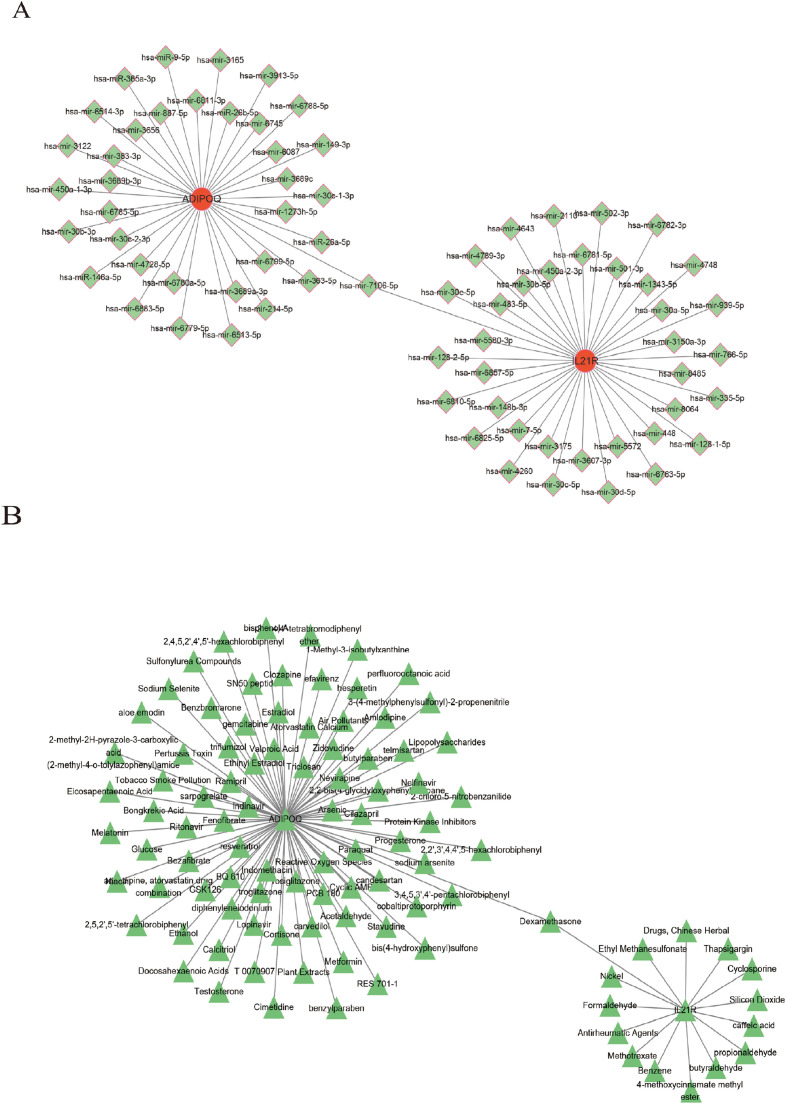
Construction of regulatory networks. **(A)** The gene-miRNA regulatory networks. **(B)** The Protein-drug/chemical interactions networks.

We constructed networks of gene-miRNA, protein-drug, and protein-chemical interactions (13), as demonstrated for the two genes in **Figure 9**. **Figure 9A** suggest that numerous miRNAs are involved in regulating these diagnostic genes, and **Figure 9B** highlights many drugs with therapeutic relevance to these genes. This provides a potential theoretical foundation for future research and targeting strategies.n

## Discussion

This study expands upon the growing body of evidence linking immune responses to cerebrovascular diseases by focusing on the role of autoimmune-related genes (ARGs) in intracranial aneurysm (IA) pathogenesis. Leveraging bioinformatics analysis and machine learning, we identified significant dysregulation of ARG expression within IA tissues compared to controls, suggesting a potential role for autoimmune responses in IA development.

In addition, elevated levels of inflammatory cytokines and chemokines, such as Tumor Necrosis Factor-α (TNF-α), Interleukin-6 (IL-6) (14, 15), and Transforming Growth Factor-β (TGF-β) (16), were detected in the blood and cerebrospinal fluid of patients with IA. The immune system undergoes significant changes following subarachnoid hemorrhage (SAH), potentially impacting patient prognosis and contributing to complications (17). These immune responses play a crucial role in the pathophysiology of SAH. A recent study by Qiaoying Li (18) investigated the immune landscape of SAH and demonstrated that an SVM classifier based on nine DEGs could effectively identify SAH patients. Using bioinformatics and statistical analysis, Dan-Dan Xu have shown that CD6 and CCR7 in inflammation-related signaling pathways are closely associated with IA rupture and may play an important role in its pathogenesis (19).This highlights the importance of understanding the immune system’s role in SAH and its potential as a diagnostic and therapeutic target. In one study that compared ruptured with unruptured IA, the expression of membrane attack complex (C activation end product) was greater in ruptured samples and associated significantly with aneurysm wall degeneration and inflammatory cell infiltration (20). Others research showed that pyroptosis is closely related to the formation and rupture of IA, and identified three potential hub genes involved in the pyroptosis and infiltration of cells (17).

We identified 39 DEARGs, including 11 upregulated and 28 downregulated genes, highlighting a disruption of immune-related processes in IA (**Figure 3A**). Functional enrichment analyses revealed these DEARGs are involved in critical biological processes and pathways, such as muscle contraction, regulation of blood circulation, cytokine-cytokine receptor interaction, and ECM-receptor interaction (**Figure 4**). These observations align with previous research demonstrating the complex interplay between structural abnormalities and immune responses in IA pathogenesis (1, 2). Notably, the enrichment of DEARGs in pathways associated with autoimmune disorders like rheumatoid arthritis further strengthens the potential link between autoimmunity and IA development (8).

Machine learning algorithms, including LASSO logistic regression, RF, and XGBoost, played a crucial role in pinpointing ADIPOQ and IL21R as potential diagnostic biomarkers for IA. The consistent selection of these genes across all three methods underscores their potential relevance. While ADIPOQ, encoding adiponectin, is primarily recognized for its metabolic functions (3), it has also been associated with synovitis and chondrocyte apoptosis in osteoarthritis (21), highlighting its broader role in inflammatory conditions. Growing evidence suggests that ADIPOQ is involved in inflammatory responses and vascular remodeling, processes directly implicated in the development of intracranial aneurysms (IA).

Similarly, IL21R, encoding the interleukin-21 receptor, is crucial for immune cell development and function, particularly in T cell differentiation and B cell activation (9). Dysregulation of IL21R signaling could contribute to an imbalance in immune responses, potentially fostering an inflammatory environment that promotes IA progression (10).

The predictive neural network model based on ADIPOQ and IL21R expression demonstrated excellent diagnostic capabilities, achieving an AUC of 0.944 (**Figure 8**). This robust performance, further validated by a nomogram approach, highlights its potential for IA risk assessment and early detection. The integration of these genetic biomarkers with clinical features in the nomogram could pave the way for personalized IA diagnosis and management.

Furthermore, our immunological analysis revealed distinct immune infiltration patterns in IA tissues, marked by increased infiltration of activated B cells, central memory CD4 T cells, and regulatory T cells (**Figure 1A**). This altered immune landscape suggests a dynamic interplay of immune responses within IA, potentially contributing to both protective and detrimental effects. The positive correlation between IL21R expression and activated CD4 T cells and activated B cells further supports its role in IA-associated immune dysregulation (22).

Our GSEA analysis provided a deeper understanding of the functional roles of ADIPOQ and IL21R in IA (**Figure 7**). ADIPOQ showed significant enrichment in immune regulatory pathways, including B cell receptor signaling and autoimmune thyroid disease, suggesting a potential role in modulating immune responses within the IA microenvironment. IL21R, on the other hand, exhibited strong associations with pathways linked to immune cell cytotoxicity, implicating its involvement in immune surveillance mechanisms within IA.

In addition, further analysis of these signature genes, including exploring their immune correlation and their interaction network with miRNAs, drugs, and chemical relationships, and other regulatory factors, could provide us with directions for subsequent targeting and immunotherapy of IA. In the future, we will continue to explore their potential mechanisms of action in IA through molecular biology experiments.

This study provides a comprehensive exploration of the molecular mechanisms underlying intracranial aneurysms (IA) using advanced bioinformatics and machine learning techniques. By integrating three independent GEO datasets, we successfully identified two key immune-related biomarkers, ADIPOQ and IL21R. However, this study has several limitations that warrant consideration. (1) Although ADIPOQ and IL21R were identified as key biomarkers, the absence of *in vitro* and *in vivo* functional experiments limits the ability to confirm their specific roles in IA pathogenesis. Functional studies are critical for validating their involvement in vascular remodeling, immune cell infiltration, and inflammatory processes. (2) The sample size of the GEO datasets used in this study is relatively small, and the data were derived from single-center studies. This may reduce the generalizability of the findings and affect the robustness of the predictive model. Larger, multicenter datasets are needed to validate and strengthen the applicability of these results. (3) While this study identified potential therapeutic targets and biomarkers, their clinical relevance and applicability remain untested. Further research is required to translate these findings into non-invasive diagnostic tools or therapeutic interventions for IA patients. (4) The regulatory networks of miRNAs and protein-drug interactions identified in this study remain theoretical. Experimental validation is essential to confirm these interactions and establish their potential as therapeutic strategies. Therefore, further experiments or trials are needed to validate these results.

## Conclusion

This study elucidates the significant role of autoimmune-related genes in the pathogenesis of intracranial aneurysms. Through comprehensive bioinformatics and machine learning analyses, including LASSO regression, RF, and XGBoost methods, we identified two key ARGs, ADIPOQ and IL21R, which serve as potential diagnostic biomarkers and offer avenues for future therapeutic interventions. Our findings lay the groundwork for future research into novel diagnostic tools and therapeutic approaches aimed at improving patient outcomes in IA.

## Data Availability

The original contributions presented in the study are included in the article/supplementary material. Further inquiries can be directed to the corresponding authors.
